# Primary Central Nervous System Vasculitis (PCNSV): A Case Report Emphasizing Diagnostic Precision and Therapeutic Approaches

**DOI:** 10.7759/cureus.74132

**Published:** 2024-11-21

**Authors:** Hiroyuki Tokue, Azusa Tokue, Issei Takahashi, Noriyuki Tagami, Yoshito Tsushima

**Affiliations:** 1 Department of Diagnostic Radiology and Nuclear Medicine, Graduate School of Medicine, Gunma University, Maebashi, JPN

**Keywords:** central nervous system inflammation, magnetic resonance imaging, meningeal enhancement, primary central nervous system vasculitis, vessel wall imaging

## Abstract

This report presents a case of primary central nervous system vasculitis (PCNSV), emphasizing the need for precise diagnosis and individualized treatment. PCNSV is a rare inflammatory condition confined to the central nervous system (CNS) that affects small- to medium-sized vessels and can cause severe neurological damage if left untreated. A 73-year-old woman with no previous medical history presented with rapidly progressive right-sided hemiparesis and cognitive impairment. Magnetic resonance imaging (MRI) findings revealed new hyperintense lesions on diffusion-weighted imaging (DWI), meningeal enhancement, and vascular wall thickening, raising suspicion of vasculitis. Cerebral angiography showed left middle cerebral artery (MCA) stenosis, and a brain biopsy confirmed perivascular lymphocytic infiltration, supporting the diagnosis of PCNSV. High-dose corticosteroids and azathioprine were administered to stabilize the symptoms. This case highlights the diagnostic value of MRI findings in PCNSV and the importance of histopathological confirmation and immunosuppressive therapy in managing this condition.

## Introduction

Primary central nervous system vasculitis (PCNSV) is a rare inflammatory condition confined to the central nervous system (CNS), characterized by inflammation of small- to medium-sized vessels. If left untreated, PCNSV can lead to irreversible neurological damage, leading to high morbidity and mortality [1]. Due to its nonspecific clinical presentation, PCNSV can be difficult to distinguish from other CNS pathologies, such as reversible cerebral vasoconstriction syndrome (RVCS), infections, and demyelinating diseases [2]. The proposed diagnostic criteria serve as the foundation for identifying PCNSV, emphasizing unexplained neurological deficits, definitive CNS vasculitis evidence via angiography or histopathology, and the exclusion of systemic vasculitis or other causative conditions [3]. Recent advances in imaging techniques and increased awareness of RVCS have led to modifications in these criteria to increase their diagnostic specificity [4]. This report describes a case of rapidly progressive PCNSV in an elderly patient, emphasizing diagnostic and therapeutic approaches and highlighting key imaging and histological findings that are essential for differentiating PCNSV from similar conditions.

## Case presentation

**Figure 1:**
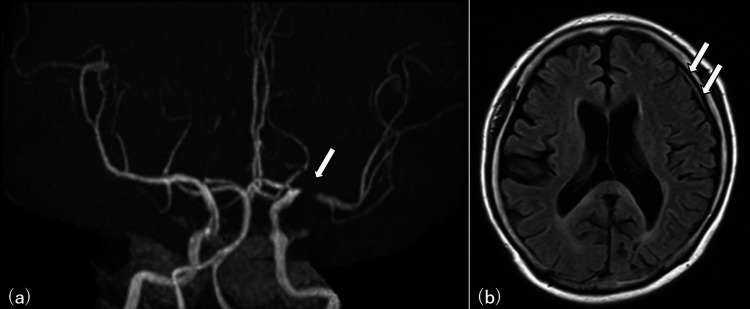
MRI one month prior to admission MRI: magnetic resonance imaging (a) Magnetic resonance angiography (MRA) revealed left middle cerebral artery (MCA) stenosis (arrow). (b) Fluid attenuated inversion recovery (FLAIR) showed dural thickening in the left cerebrum (arrows)

A 73-year-old woman with no previous medical history presented with visual field disturbance. One month before admission, she developed right homonymous inferior quadrantanopia, and brain magnetic resonance imaging (MRI) revealed stenosis of the left middle cerebral artery (MCA) and dural thickening (Figure 1).

**Figure 2:**
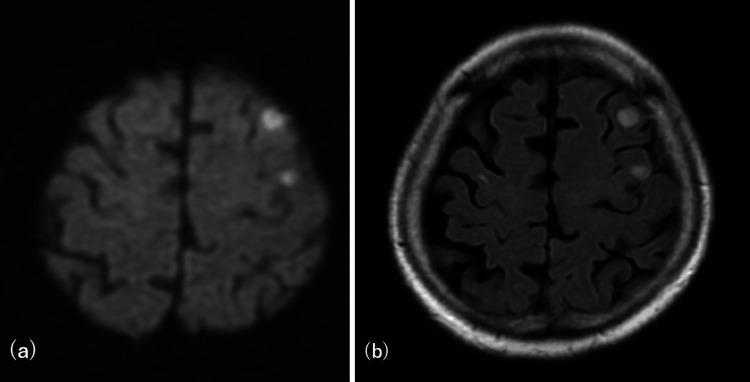
MRI two weeks prior to admission. Hyperintense lesions appeared on the left frontal lobe MRI: magnetic resonance imaging (a) Diffusion-weighted imaging (DWI). (b) Fluid attenuated inversion recovery (FLAIR)

Two weeks prior to admission, new hyperintense lesions appeared in the left frontal lobe on diffusion-weighted imaging (DWI), which were initially thought to be ischemic (Figure 2).

**Figure 3:**
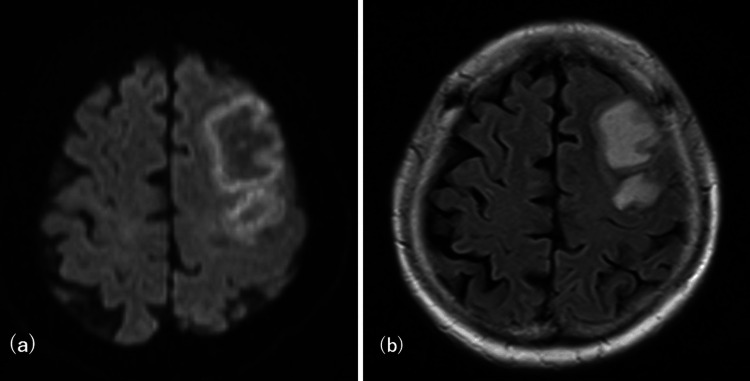
MRI on admission showed lesion expansion MRI: magnetic resonance imaging (a) Diffusion-weighted imaging (DWI). (b) Fluid attenuated inversion recovery (FLAIR)

**Figure 4:**
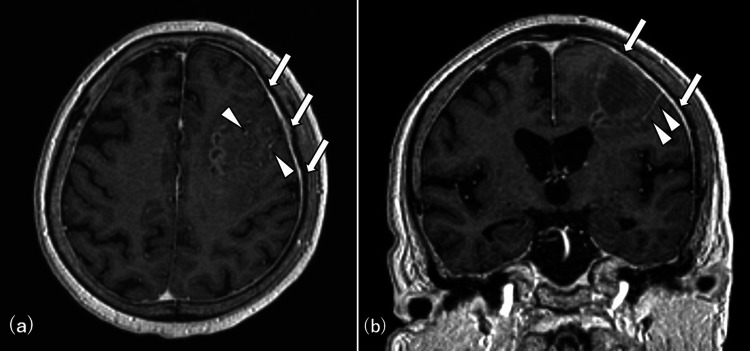
MRI showed meningeal enhancement (arrows) and vascular wall thickening (arrowheads) on gadolinium-enhanced T1-weighted imaging MRI: magnetic resonance imaging (a) Axial image. (b) Coronal image

However, her symptoms worsened, prompting a referral for advanced evaluation. On admission, neurological examination revealed motor aphasia, right-sided hemineglect, and a Glasgow Coma Scale score of E4V4M6. The laboratory test results, including those for autoimmune and infectious markers, were unremarkable. Cerebrospinal fluid (CSF) analysis showed no abnormalities, with a cell count of 1/µL, protein level of 29 mg/dL, and normal glucose. MRI revealed lesion expansion on DWI/fluid attenuated inversion recovery (FLAIR) (Figure 3), along with meningeal enhancement and vascular wall thickening on gadolinium-enhanced sequences, raising the suspicion for vasculitis (Figure 4).

**Figure 5:**
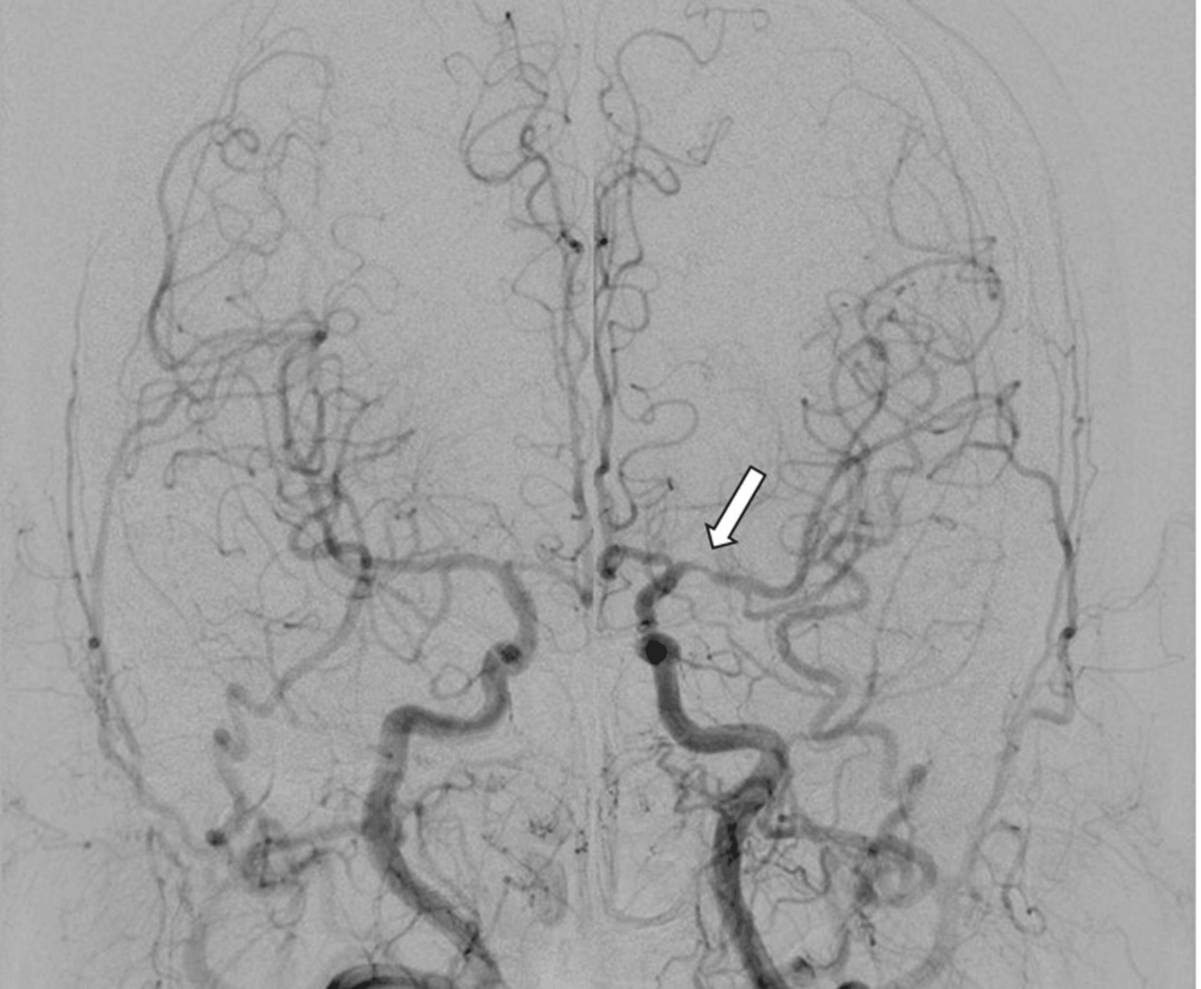
Digital subtraction angiography confirmed left middle cerebral artery stenosis (arrow)

Blood tests for cANCA/PR3 and pANCA/MPO were performed to rule out differential diagnoses such as granulomatosis with polyangiitis. The results of these tests were normal. Angiography confirmed left MCA stenosis (Figure 5).

**Figure 6:**
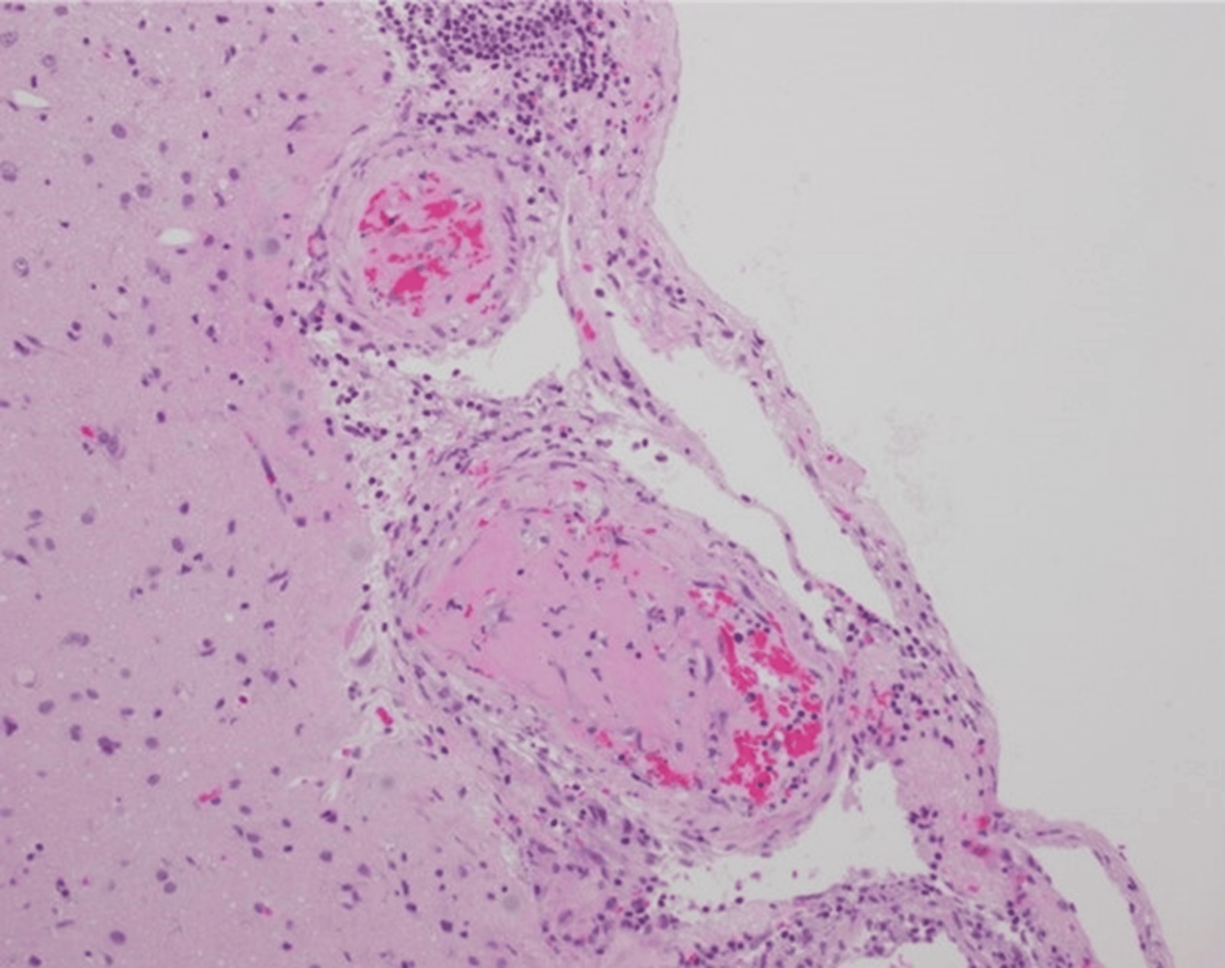
Biopsy samples showed vasculitis with thrombosis and cerebral ischemic lesion Hematoxylin and eosin stained; original magnification, ×200

Brain biopsies from both ischemic and nonischemic cores revealed perivascular lymphocytic infiltration in small vessels (Figure 6), consistent with PCNSV and ruling out infection, demyelination, RVCS, or amyloid angiopathy.

Postbiopsy treatment and outcome

High-dose corticosteroid pulse therapy (Prednisolone, 1 mg/kg/day) was initiated and continued for two weeks, leading to initial symptomatic improvement. However, during the tapering phase over the subsequent week, the patient’s condition worsened, with the emergence of new cerebral lesions. To address this, azathioprine (Imuran, 1 mg/kg/day) was introduced as an adjunct to corticosteroid therapy. Azathioprine therapy was maintained for two months to facilitate controlled tapering of corticosteroids while ensuring disease control. This combination eventually led to symptom stabilization and reduced steroid-related complications, such as steroid-induced diabetes, which is particularly concerning in elderly patients. After stabilization, the patient was transferred to a rehabilitation facility for further care.

## Discussion

This case illustrates the diagnosis and management complexities of PCNSV, which is a rare but severe form of CNS vasculitis. The diagnostic criteria proposed by Calabrese and Mallek in 1988 provided a foundational framework for identifying PCNSV, emphasizing the following: (a) persistent, unexplained neurological deficits despite extensive evaluation, (b) definitive CNS vasculitis evidenced by angiographic or histopathological findings, and (c) exclusion of systemic vasculitis or other potential causes of these findings [3].

A critical aspect of diagnosing PCNSV is distinguishing it from RVCS because both conditions can present with similar clinical and angiographic findings. In 2009, Birnbaum and Hellmann proposed revising the diagnostic criteria to address this challenge [4]. According to the updated criteria, a definitive diagnosis of PCNSV requires histopathological confirmation through CNS biopsy, as was achieved in this case. When biopsy is not feasible, a probable diagnosis of PCNSV can be made based on high-probability angiographic findings, abnormal MRI results, and a CSF profile consistent with PCNSV [4-6]. This stratified approach helps avoid misdiagnosis and inappropriate cytotoxic treatment in patients with RVCS, a self-limiting condition that does not require aggressive immunosuppressive therapy [4-6].

In this case, CSF analysis showed normal. It has been reported that CSF analysis for PCNSV showed abnormalities such as mild cell count and elevated protein in the acute phase in 80%-90% of cases, with no specific changes. CSF analysis is often performed to rule out infection [7]. Moreover, histopathological confirmation led to a definitive diagnosis of PCNSV, ruling out RVCS and other potential CNS pathologies. While PCNSV lacks pathognomonic imaging features, MRI abnormalities are present in nearly all cases and are essential for diagnosis. Common MRI findings include cortical and subcortical infarcts, intracerebral hemorrhages, meningeal (leptomeningeal) enhancement, vessel wall enhancement, and T2WI-hyperintense lesions in the cerebral white matter [7,8]. In this patient, meningeal enhancement and vessel wall thickening raised suspicion of PCNSV, underscoring the diagnostic value of MRI in identifying these specific features. The presence of cortical infarcts and meningeal involvement helped further differentiate PCNSV from RVCS, as RVCS typically presents with transient, reversible vasoconstriction and is less likely to cause persistent parenchymal changes.

Reports of dural thickening in PCNSV are rare. Although dural thickening is commonly seen in other vasculitis types, such as granulomatosis with polyangiitis (GPA), eosinophilic GPA (EGPA), and Behçet’s disease, it is not typically observed in PCNSV [9]. In our patient, we hypothesized that the dural thickening was a secondary manifestation of inflammation in the superficial cerebral arteries. Recognizing these atypical presentations is essential, as they provide further insights into the inflammatory patterns of PCNSV and help differentiate it from other types of CNS vasculitis.

Histopathological confirmation remains the gold standard for the definitive diagnosis of PCNSV, as demonstrated in this case. However, biopsy is often reserved for cases where imaging is inconclusive or differentiation from other CNS conditions is challenging. In this case, the combination of imaging findings (cortical infarcts, meningeal enhancement, and vessel wall thickening) and histopathology enabled a definitive diagnosis of PCNSV, facilitating prompt initiation of immunosuppressive treatment.

This case also highlights the need for early and aggressive treatment of PCNSV to prevent irreversible CNS damage. Corticosteroids are generally the first-line therapy; however, in cases of corticosteroid resistance, as observed in this patient, additional immunosuppressive agents like azathioprine or cyclophosphamide may be necessary. In our patient, the addition of azathioprine allowed for gradual corticosteroid tapering, effectively stabilizing the disease progression and reducing steroid-related complications, which are particularly relevant given her advanced age.

## Conclusions

This case underscores the importance of precise diagnosis and tailored treatment in managing PCNSV, especially given its potential to mimic other CNS pathologies. Histopathological confirmation remains critical for distinguishing PCNSV from RVCS and guiding treatment decisions. Early intervention with corticosteroids and adjunctive immunosuppressive therapy has proven effective in stabilizing disease progression, emphasizing the need for prompt and accurate diagnostic and therapeutic approaches in PCNSV management.
